# Comparative secretome analysis reveals cross-talk between type III secretion system and flagella assembly in *Pseudomonas plecoglossicida*

**DOI:** 10.1016/j.heliyon.2023.e22669

**Published:** 2023-12-02

**Authors:** Pan Qin, Yingjia Luan, Jinmei Yang, Xingfu Chen, Tong Wu, Yousheng Li, Hetron Mweemba Munang'andu, Guangming Shao, Xinhua Chen

**Affiliations:** a Key Laboratory of Marine Biotechnology of Fujian Province, College of Marine Sciences, Fujian Agriculture and Forestry University, Fuzhou, 350002, China; b Southern Marine Science and Engineering Guangdong Laboratory (Zhuhai), Zhuhai, 519000, China; c Faculty of Biosciences and Aquaculture, Nord University, Bodø, 8026, Norway

**Keywords:** *Pseudomonas plecoglossicida*, Secretome, Type III secretion systems, *popBD*, Flagella

## Abstract

The Gram-negative bacterium *Pseudomonas plecoglossicida* has caused visceral granulomas disease in several farmed fish species, including large yellow croaker (*Larimichthys crocea*), which results in severe economic losses. Type III secretion systems (T3SS) are protein secretion and translocation nanomachines widely employed by many Gram-negative bacterial pathogens for infection and pathogenicity. However, the exact role of T3SS in the pathogenesis of *P. plecoglossicida* infection is still unclear. In this study, a T3SS translocators deletion strain (△*popBD*) of *P. plecoglossicida* was constructed to investigate the function of T3SS. Then comparative secretome analysis of the *P. plecoglossicida* wild-type (WT) and △*popBD* mutant strains was conducted by label-free quantitation (LFQ) mass spectrometry. The results show that knockout of T3SS translocators popB and popD has an adverse effect on the effector protein ExoU secretion, flagella assembly, and biofilm formation. Further experimental validations also confirmed that *popB-popD* deletion could affect the *P. plecoglossicida* flagella morphology/formation, adherence, mobility, and biofilm formation. These data indicate that a cross-talk exists between the *P. plecoglossicida* T3SS and the flagella system. Our results, therefore, will facilitate the further under-standing of the pathogenic mechanisms leading to visceral granulomas disease caused by *P. plecoglossicida*.

## 1 Introduction

*Pseudomonas plecoglossicida* is a Gram-negative bacterium that generally inhabits the soil and sediments in aquatic environments. Multiple studies have shown that *P. plecoglossicida* plays an important role as a biological factor in fish disease outbreaks [[1], [2], [3], [4], [5]]. Given its severity linked to high mortalities in several farmed fish species that include large yellow croaker (LYC) (*Larimichthys crocea*) [2,6], ayu (*Plecoglossus altivelis*) [7], Orange spotted grouper (*Epinephelus coioides*) [8], rainbow trout *(Oncorhynchus mykiss*) [9] and barramundi (*Lates calcarifer*) [10], the need to elucidate its pathogenic mechanism has attracted a lot of research interests in an effort to develop effective control measures against the visceral white spot disease caused by *P. plecoglossicida.* Like all pathogenic bacteria, its pathogenicity largely depends on the virulence factors encoded in its genome. Thus, to fully understand its pathogenic mechanisms, it is important to elucidate the functional roles of various virulence factors encoded in *P. plecoglossicida*. Among virulence factors, secretion systems and the effectors transported through them are especially relevant [[11], [12], [13], [14]].

In 2013, the draft genome of *P. plecoglossicida* strain NB2011 was reported [15]. There were several virulence factors that were discovered, including the type III (T3SS) and type VI (T6SS) secretion systems, as well as flagellar system [15]. These findings have paved the way for studies aimed at elucidating the role of various genes in the pathogenicity of *P. plecoglossicida.* As for the T6SS genes, a recent study showed that ClpV plays a vital role in the pathogenicity of *P. plecoglossicida* in Orange spotted grouper [16]. At the same time, another study has demonstrated that T6SS genes are critical for granuloma formation [17], in line with other studies [18]. In the case of flagella genes, silencing the *flgK* and *fliG* genes drastically attenuated the mobility, adhesion, and biofilm formation of *P. plecoglossicida* and the reduced bacterial loads, reduced granuloma formation and high post-challenge survival were observed [19,20]. However, the role of the T3SS in *P. plecoglossicida* is less well-studied than the T6SS and flagella.

The T3SS is a conserved syringe-like structure which is composed of a basal body, an external needle, and a translocon [21]. To establish infection, the bacterium injects effector proteins through the T3SS into the host cell cytosol. The effectors are crucial for bacterial pathogenesis as they can manipulate the host cell by hijacking signaling pathways [22]. In *P. aeruginosa*, proteins that form the translocon include the hydrophobic translocators popB and popD, and the hydrophilic translocator PcrV [23,24]. The PcrV tip is responsible for sensing the host cell membrane [25]. Upon contact of the PcrV with the host cell membrane, popB is inserted into the membrane, resulting in a conformational change in the tip and secretion activation [26]. However, as of now, it remains unclear how secretion is prevented until the T3SS receives an activation signal and how this signal is communicated to the cytoplasmic side to activate it. Evidences show that popB and popD like translocon proteins found in other bacteria species play key roles in blocking and controlling secretion in the absence or presence of a secretion inducing signal. For example, Veenendaal et al. [27] and Menrad et al. [28] showed that the translocon IpaB and IpaD proteins of *Shigella flexneri*, which are the homology of popB and popD, are essential for preventing the leakage of cytoplasmic proteins into extracellular compartments. Thus, the disruption or deletion of translocon proteins leads to leakage of cytoplasmic proteins into extracellular compartments. As such, deposition of cytoplasmic proteins in extracellular compartments due to defective T3SS translocon proteins have been reported in different bacteria species [[29], [30], [31], [32]]. Thus, translocon proteins have a dual purpose of preventing extracellular leakage of cytoplasmic proteins and intracellular deposition of toxins after penetrating the host cell membrane.

Although a 24-kb gene cluster that possibly encoded the T3SS system was observed in *P. plecoglossicida* (NB2011) [15], the functional roles of these genes are yet to be determined. Zhang et al. have identified a typical T3SS effector (ExoU) in *P. plecoglossicida*, which has a conserved phospholipase A (PLA) domains at their *N*-terminal with other homologs [12]. They also demonstrated that ExoU was expressed and secreted *in vitro* in the wild strain but not expressed in ExsA-deleted mutant, indicating ExsA is required for the *P. plecoglossicida* T3SS effector secretion [12]. In most Gram-positive bacteria, the T3SS effectors secretion is usually induced by contact with the host cell or is induced by a low calcium concentration or other factors *in vitro* [21]. Interestingly, the *P. plecoglossicida* T3SS effector ExoU can be secreted into supernatants in LB broth and Minimum Essential Medium (MEM) without EGTA, indicating that EGTA induction cannot significantly enhance *P. plecoglossicida* ExoU secretion [12].

Different virulence factors do not occur in isolation but are closely interconnected. For example, the functional connection between the T6SS and flagella [33,34], and the linkage between T3SS and T6SS [35,36] have been observed in some bacteria. However, the interplay between T3SS and the flagella system has not been explored in *P. plecoglossicida*. Thus, it is unknown whether the coexistence of the T3SS and flagella proteins potentiate each other or if they are antagonistic in the virulence of *P. plecoglossicida*. These informational gaps warrant further studies aimed at elucidating the role of the T3SS, and its relationship with flagella, in the virulence of *P. plecoglossicida*.

In this study, we developed a T3SS translocon depletion mutant (Δ*popBD*) of *P. plecoglossicida* with the view to (i) compare the profile of differentially secreted proteins (DSPs) between the Δ*popBD* mutant, and wild-type (WT) strains, (ii) identify the network pathways affected by *popB-popD* deletion, (iii) explore the impact of Δ*popBD* knockout on the flagella morphology/formation, adherence, mobility, and biofilm formation, and (iv) determine whether there is a cross-talk between the T3SS machinery and flagella assembly. Overall, we anticipate that the data presented here will shed new insights into the pathogenic mechanisms of *P. plecoglossicida* and contribute to the design of protective vaccines.

## 2 Materials and methods

### 2.1 Bacterial strains, plasmid, and growth conditions

*P. plecoglossicida* strain PQLYC4 [3] was used to generate the mutant strain described in this study. The bacteria were grown in tryptic soy broth (TSB) or tryptic soy agar (TSA) plates at 28 °C. To prepare the secretome samples, *P. plecoglossicida* strains were cultured at 28 °C in an M9 minimal medium.

### 2.2 Molecular cloning and construction of bacterial mutants

*P. plecoglossicida* T3SS structure protein deletion mutants (Δ*pop BD*) were generated by homologous recombination described as [37,38] with some modifications. The pEX18Tc sucrose counter-selection system was used for unmarked gene deletion. Briefly, the upstream and downstream fragments of *popB* and *popD* (18Tc-Δ*popBD*-UP F; 18Tc-Δ*popBD*-UP R; 18Tc-Δ*popBD*-down F; 18Tc-Δ*popBD*-down R) were amplified with the primers listed in Table 3. The upstream and downstream fragment was cloned into the vector pEX18Tc yielding recombinant pEX18Tc-*popBD* up-down plasmid and transformed into *E. coli* S17-1λ. After sequence validation, the colony was inoculated in an LB medium containing 100 μg/mL tetracycline. The knockout mutant strains were obtained through biparental mating. After overnight culture, the donor strain *Escherichia coli* containing the plasmid pEX18Tc-*popBD* up-down in LB and the recipient strain *P. plecoglossicida* grown in TSB were cultured, and then collected and resuspended in PBS. The bacteria were mixed in a 1:2 ratio and then spotted them onto LB agar plates. Bacteria were scraped off and resuspended in 500 mL of TSB after being culturing at 28 °C for 24 h. In order to select for merodiploids, the diluted suspensions were spread on TSA plates containing 100 mg/mL tetracycline. A double crossover was determined by streaking the grown colony on a TSA plate containing 15 % sucrose after the first crossover. The resultant *popBD* knockout mutant was verified with the primers targeting the knockout (internal) sequence or upstream and downstream of the knockout sequence (external) listed in Table 3.

To construct *popBD* complementary strain, *popB* and *popD* genes were amplified by PCR and ligated to pMMB67 E H with restriction enzyme *Eco*Rl and *Pst*I sites. After sequence verification, the recombinant plasmid was introduced into the Δ*pop BD* strain by electroporation as previously described [39].

### 2.3 Determination of the bacterial growth curves

*P. plecoglossicida* WT and △*popBD* mutant strains were grown on TSB medium overnight at 28 °C. The next day, Cultures were diluted in TSB medium to a starting optical density of 600 nm (OD_600nm_) of 0.05 and incubated at 25 °C, and OD_600 nm_ values were determined every hour for 30 h. The experiment was performed in three duplicates.

### 2.4 Generation of ExoU protein and *anti*-ExoU polyclonal antibodies

The ExoU ORF was cloned into the pET-28a (+) vector (Novagen). After DNA sequencing validation, the recombinant plasmid was transformed into *Rosetta* (DE3) competent cells (Transgen biotech, China), and induced for 6 h with 1 mM IPTG. A supersonic schizolysis method was used to lyse bacteria, which were collected by centrifugation at 8000 rpm for 15 min. The supernatants and precipitates were analyzed by the SDS-PAGE. Then the purified ExoU protein was used to prepare *Anti*-ExoU polyclonal antibodies by immunizing New Zealand white rabbits as previously described [40].

### 2.5 Preparation of bacterial culture supernatants

10 mL of *P. plecoglossicida* WT or △*popBD* mutant culture were incubated for 12 h (exponential phase) and 24 h (stationary phase), shaking at 200 rpm at 28 °C. After incubation, the culture supernatants secreted by *P. plecoglossicida* were collected. Afterward, the sample was passed through a 0.22 μm membrane filter to ensure that no intact bacteria remained. The protein was quantified using a bicinchoninic acid (BCA) Protein Assay Kit (Bio-Rad, Hercules, CA, USA). For each sample, the equal amount of protein was mixed with loading buffer and boiled for 5 min. The proteins were separated on 12.5 % SDS-PAGE gel and detected with *anti*-ExoU polyclonal antibodies.

### 2.6 Protein extraction and digestion

Proteins in culture supernatants were precipitated with 6 % (w/v) trichloroacetic acid (TCA, Sigma), collected after centrifuging at 15,000×*g* for 15 min at 4 °C, and washed twice with ice-cold 80 % (v/v) acetone. Then the precipitate samples were solubilized in SDT buffer for sample lysis and protein extraction. The protein was quantified using a bicinchoninic acid (BCA) Protein Assay Kit (Bio-Rad, Hercules, CA, USA). The filter-aided sample preparation (FASP) method was used to digest proteins with trypsin. After digesting, the peptides were desalted on C18 Cartridges (Empore™ SPE Cartridges C18 (standard density), bed ID 7 mm, volume 3 mL; Sigma, St. Louis, MO, USA) and concentrated. Upon centrifugation, the solutions were reconstituted in 0.1 % (v/v) formic acid. For each sample, 20 g of protein was mixed with loading buffer and boiled for 5 min. The proteins were separated on 12.5 % SDS-PAGE gel and visualized by Coomassie Blue staining.

### 2.7 LC-MS/MS analysis

A Q Exactive mass spectrometer in conjunction with an Easy nLC were used to perform LC-MS/MS analysis. Reverse-phase trap columns connected to C18-r reversed-phase analytical columns were loaded with peptides in 0.1 % formic acid, then separated with a linear gradient of buffer B, which consist of 84 % acetonitrile and 0.1 % Formic acid, at a flow rate of 300 nl/min. The mass spectrometer was operated in positive ion mode.

MS data were acquired using a data-dependent top 10 method, dynamically choosing the most abundant precursor ions from the survey scan (300–1800 *m*/*z*) for HCD fragmentation. It was set to 3e6 for the automatic gain control (AGC) target and 10 ms for the maximum injection time. The dynamic exclusion duration set as 40.0 s. A survey scan was acquired at 70,000 *m*/*z* 200 resolution, and HCD spectra resolution were acquired at 17,500 *m*/*z* 200, with a 2 *m*/*z* isolation width. The normalized collision energy was 30 eV, and the underfill ratio was defined as 0.1 %.

### 2.8 Identification and quantitation of proteins

To identify and quantify the peptides in each sample, the raw MS data was combined and searched using MaxQuant 1.5.3.17 software (Max Planck Institute of Biochemistry in Martinsried, Germany). We identified the proteins by searching the UniProt *P. plecoglossicida* database (ID: UP000014440) which contains 4883 protein sequences. The identification parameters were set as follows: trypsin was selected as the enzyme; maximum missed cleavages set as 2; fixed modifications-carbamidomethyl; variable modifications-methionine oxidation; main search, first search and MS/MS tolerance were 6 ppm, 20 ppm and 20 ppm respectively. The false discovery rate (FDR) of protein and peptide identification was ≤0.01. For the quantified proteins, only *p*-values below 0.05 and fold changes above 2.0 were considered significant by the two-sided *t*-test.

### 2.9 Bioinformatic analysis

The amino acid sequences were searched using the NCBI database and InterProScan software. A multi-class SVM classification system, CELLO (http://cello.life.nctu.edu.tw/), was used to predict protein subcellular localization. The gene ontology (GO) terms were mapped, and sequences were annotated using the software program Blast2GO. The GO annotation results were plotted using R scripts. After annotation, a BLAST search was conducted against the KEGG database (http://geneonto logy. org/) to identify the proteins' KEGG orthology, and then mapped to KEGG pathways. Based on Fisher's exact tests, enriched analyses were performed using the whole set of quantified proteins as the background. Further adjustment of the derived p-values was made using the Benjamini-Hochberg correction. A significant functional category or pathway was defined as having a *p*-value of less than 0.05. Protein-protein interactions (PPIs) among the studied proteins were determined using STRING software (http://string-db.org/)by retrieving information from the IntAct molecular interaction database (http://www.ebi.ac.uk/intact/) [41].

### 2.10 Electronic microscopy

The pure colony of *P. plecoglossicida* WT or mutant strain was cultured in TSB at 28 °C for 12 h for morphology observation. A volume of 200 μL bacterial culture was centrifuged at 4000 rpm at 4 °C for 5 min. After discarding the supernatant, the precipitate was washed with distilled water and resuspended in 100 L of distilled water. The cell suspension was allowed to adhere to a formvar-coated copper mesh grid for 10 s, and the excess culture was drained off. In the next step, adherent cells were stained negatively with a 2 % aqueous solution of phosphotungstic acid for 30 s. The bacterial morphology was observed using a TEM (Hitachi H-7650, Hitachi High-Tech) operating at 80 kV.

### 2.11 Motility assays

Swarming motility assays were performed as previously described in Ref. [42] with slight modifications, as shown below. The *P. plecoglossicida* WT or mutant strains were inoculated in the TSB medium and incubated overnight. A sterile pipette tip was dipped into cultures, and bacterial cultures were spotted onto TSA medium with 0.5 % agar. Plates were incubated for 12, 18, and 24 h at 28 °C and the diameter across was measured. Swimming motility assays were performed as previously described [43]. We used the sterile pipette tip to stab the overnight cultured bacterial into the agar layer of the plate (M8 medium with 0.3 % agar concentration). Plates were incubated upright at 28 °C for 12, 18, and 24 h and growth was measured as the diameter across which the bacteria grew.

### 2.12 In vitro assay of bacterial adhesion onto EPC cells

*In vitro* assay of bacterial adhesion was conducted as described previously [44] with some modifications. Three sets of duplicate wells were seeded with 2 × 10^5^ cells in a 24-well plate. The cells were then washed with phosphate-buffered saline (PBS) and the culture medium replaced with antibiotic-free DMEM supplemented with 10 % FBS after overnight culture. Overnight cultures of *P. plecoglossicida* WT or mutant strain cells were collected and resuspended in M199 with 10 % FBS. 10^6^ *P. plecoglossicida* WT or mutant strain bacterium was used to inoculate EPC cells, which corresponding to a multiplicity of infection (MOI) of 5. The same volume of *P. plecoglossicida* or mutant strain culture was also added to the medium without EPC cells to determine the total number of bacterial cells in the inoculum. After 3 h of incubation, the culture medium was removed and the infected EPC cells were washed with PBS. 0.05 % trypsin-EDTA was used to detach all the cells for 20 min at 37 °C. Then, serial dilutions of these samples were plated on TSA plates, and the adherent cells were counted. The percentage of adhered bacteria was quantified by counting colony-forming units (CFU) [(Number of the detected bacterial cells/Number of bacterial cells initially added to the EPC cells) × 100 (%)]. The results are shown as the mean ± SD from three independent experiments.

### 2.13 Biofilm formation assay

The biofilm formation assay was conducted as described in Ref. [45] with some modifications. Briefly, overnight cultures of the *P. plecoglossicida* WT or mutant strains were diluted in 1:100 with TSB medium. Then, the dilutions were added into 96-well plates with 100 μL per well. After incubation for 24 h at 28 °C, the plates were gently rinsed twice with water. Each well was stained with 125 μL of 0.1 % crystal violet solution for 15 min, then allowed to dry overnight. 125 μL of 30 % acetic acid was added to each microtiter plate well to solubilize the crystal violet. After incubation for 15 min, biofilm formation was visualized by measuring absorbance at OD_550_ nm. The plates were filled with 125 μL of 30 % acetic acid in water as the blank. The results are shown as the mean ± SD from three independent experiments.

### 2.14 RNA extraction and qRT-PCR

*P. plecoglossicida* WT and △*popBD* mutant strains were grown overnight at 28 °C in the TSB medium. The next day, cultures were diluted to a starting optical density at OD_600nm_ of 0.05. Once inoculated, the tubes were placed in a 28 °C shaking incubator and left to agitate at 180 rpm for 6 and 12 h. The bacteria pellets were collected and subjected to RNA extraction with the bacterial RNA Isolation Kit (Invitrogen) according to the manufacturer's instructions. Bacterial pellets were resuspended in preheat Max Bacterial Enhancement Reagent and incubated at 95 °C for 4 min. Then, 1 mL TRIzol (Invitrogen) was added, and the suspension was incubated for 5 min at room temperature. 0.2 mL cold chloroform was added and mixed by shaking the tube vigorously. After 3 min of incubation at room temperature, the tubes were centrifuged for 15 min at 12,000×*g* at 4 °C. Then the mixture was separated into a lower red phenol-chloroform phase, interphase, and a colorless aqueous phase containing RNA. A volume of 400 μl colorless aqueous phase was transferred to a fresh tube, and 500 μl cold isopropanol was added to the aqueous phase to precipitate RNA. The solution was incubated in the tube at room temperature for 10 min and centrifuged for 10 min at 15,000×*g* at 4 °C. Nucleic acid pellets were washed with 70 % ethanol and dried by air. Chromosomal DNA was digested using the Turbo DNaseI (Ambion) kit following the manufacturer's instructions.

Transcriptional levels of flagella-related genes in *P. plecoglossicida* WT or mutant strains were determined by quantitative reverse transcription PCR (qRT-PCR) with the primer pairs listed in Table 3. Quantifying gene expression changes relative to their control group was done using the 2^−ΔΔCt^ method. All quantifications were normalized using the *rpod* reference gene, which remained constant regardless of experimental conditions. Error bars represent ±SD (n = 3).

## 3 Results

### 3.1 Effect of the knockout of the popB and popD gene on the growth and protein secretion

**Fig. 1:**
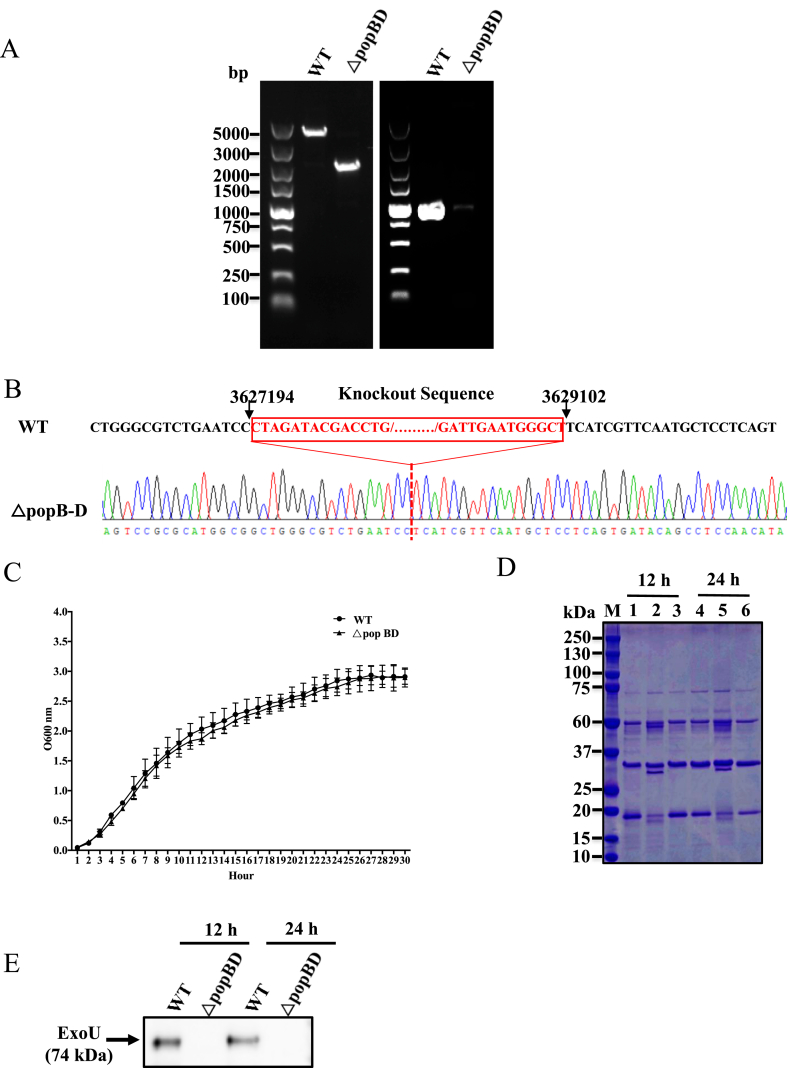
(A) Confirmation of *popBD* gene knockout by PCR with pairs of primers designed to target within or outside of the *popBD* genes. M, Marker DL5000; (B) The map of the sequencing of PCR amplification products and alignment result showing 1904 bp deletions in △*popBD* strain; (C) Characterization of the bacterial growth curves of *P. plecoglossicida* WT and △*popBD* mutant strains. There were no significant differences in bacterial growth curves of the WT and Δ*popBD* mutant strains of *P. plecoglossicida* measured as OD_600_nm values at 1 h intervals. The data are presented as means of three replicates. Error bars represent standard deviations. (D) Secretion profiles of the *P. plecoglossicida* WT, △*popBD* mutant and complementary strains. 20 μg supernatant protein samples from the WT (lane 1, 12 h; lane 4, 24 h), *△popBD* mutant (lane 2, 12 h; lane 5, 24 h) and complementary strain (lane 3, 12 h; lane 6, 24 h) grown in the M9 minimal medium were concentrated and separated using SDS-PAGE. (E) *P. plecoglossicida* ExoU secretion and Western blotting detection. The supernatant of *P. plecoglossicida* WT and △*popBD* mutant strains cultured in MEM were probed by Immunoblotting using *anti*-ExoU polyclonal antibody.

The T3SS basal body, needle filament, and translocation apparatus are essential for the translocation of the effector into host cells. In this study, we constructed T3SS function-deficient strains by knocking out the genes encoding the translocation apparatus (*popB*, *popD*). Gene knockout was firstly verified by colony PCR using specific primer pairs targeting the knockout domain (internal primer) or the upstream and downstream regions of the domain (external primer). Using the external primer targeting the knockout domain, a fragment deleted could be observed in the mutant strain (Fig. 1A, left). Using the internal primer, an 887-bp band was amplified from the genomic DNA of the WT strain, but no band was found in the mutant strains (Fig. 1A, right). We further confirmed that the *popBD* gene had been successfully deleted by sequencing (Fig. 1B).

As mutations by deletion might cause growth defects, the WT and Δ*popBD* strains were cultured in a TSB medium and monitored by optical density measurement. There were no significant differences in growth between the WT and mutant strains, demonstrating that the knockout of *popB* and *popD* genes did not cause any noticeable growth defect (Fig. 1C). Interestingly, compared to the WT strain, more proteins were detected in the supernatant of the Δ*popBD* mutant strain.

To quantitatively evaluate the effect of *popBD* knockout on bacterial protein secretion, equal quantities (20 μg) of spent medium samples obtained from the WT and mutant bacterial cultures were analyzed by SDS-PAGE followed by Coomassie staining. As shown in Fig. 1D, the secreted protein profile of the WT strain is different from the profile of the mutant strains at 12 h and 24 h. Furthermore, a *popBD* complemented strain was recovered in similar quantity to WT.

We also detected the WT and mutant strain T3SS effector ExoU secretion by Western blotting. The result showed that, compared to the WT, the target band was not detected in supernatants of the mutant strain cultured under similar conditions (Fig. 1E), indicated that the *popBD* knockout impaired the function of the T3SS.

### 3.2 Comparative secretome analysis between WT and the △popBD knockout mutant strains

To investigate the effect of *popB* and *popD* knockout on protein secretion in *P. plecoglossicida*, the total proteins in the supernatant from the WT and mutant strains (Δ*popBD*) were extracted and quantified using a label-free quantitative proteomic strategy. As shown in Table 1, WT compared with the Δ*popBD* strain, 82 and 89 proteins were differentially secreted (*p* < 0.05, fold change>2) at 12 h (59 upregulated, 23 downregulated) and 24 h (59 upregulated, 30 downregulated). Besides, some proteins were identified in at least two of three replicates in one group, while another group had all null values. 29 and 19 proteins were detected in the supernatant of *P. plecoglossicida* WT but not in the Δ*popBD* strain at 12 and 24 h, respectively. At the same time, 735 or 1064 proteins were detected only in the supernatant of Δ*popBD* strain at 12 and 24 h, respectively (Supplemental Table 1). More proteins were identified in the supernatant of △*popBD*, which might be due to leakage of the proteins after the T3SS translocator deletion.

**Table 1:** Summary of differential secreted proteins identified in the *P. plecoglossicida* WT versus △*popBD* mutant strains.

Comparisons	Significantly changing in abundance^a^		Consistent presence/absence expression profile^b^	
	Upregulated	Downregulated	WT strain	Mutant strain
12 h	59	23	29	735
24 h	59	30	19	1064

a Fold change>2.0 and *p*-value<0.05.

b Proteins identified in at least two of three replicates in one group while another group with all null value.

### 3.3 Functional characterization and enrichment of differentially quantified proteins

**Fig. 2:**
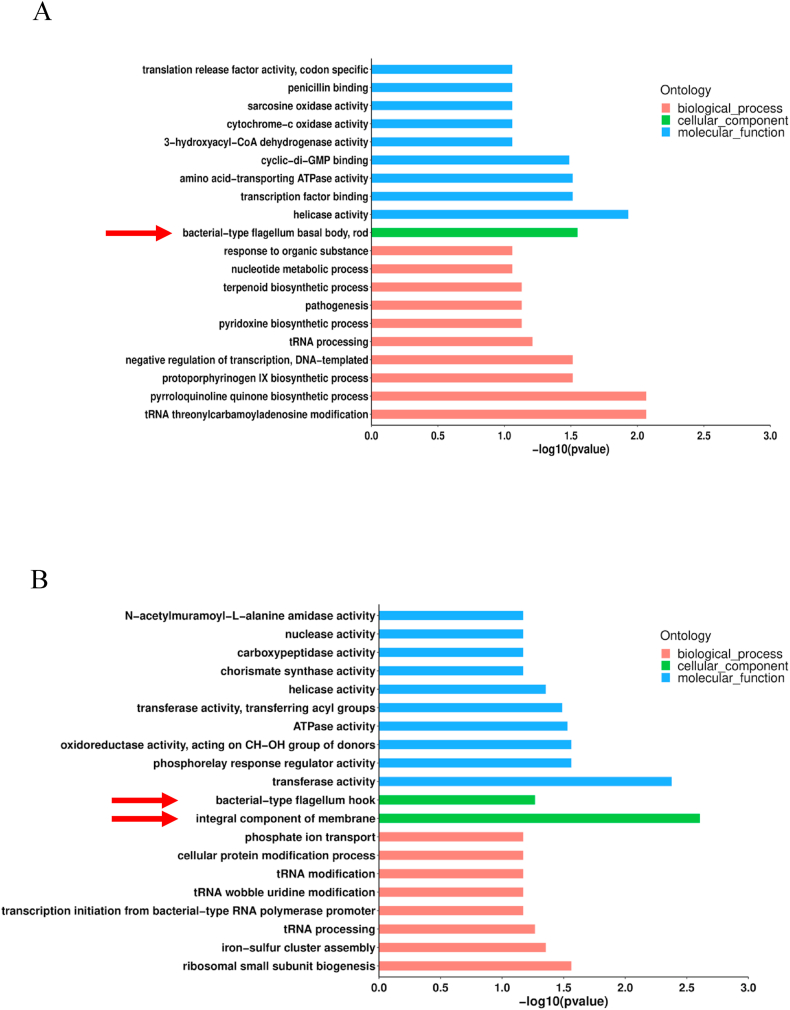
GO-based enrichment analysis of the identified DSPs for 12 h (A) and 24 h (B). A two-tailed Fisher's exact test was used to test the enrichment of the identified proteins in each category against all database proteins.

To obtain functional information about the GO and KEGG analysis was performed on the upregulated and downregulated proteins with *p*-values less than 0.05 taken as significant. Gene ontology (GO) terms were mapped and sequences were annotated using the software program Blast2G.The top 20 enrichment of DSPs from WT versus Δ*popBD* were mapped to biological process, molecular function, and cellular component (Fig. 2A and B). Of concern is that the most enriched term in the cellular component category was the bacterial-type flagellum basal body, rod at 12 h (Fig. 2A, red arrow), bacterial-type flagellum hook, and the integral component of the membrane at 24 h (Fig. 2B, red arrow).

**Fig. 3:**
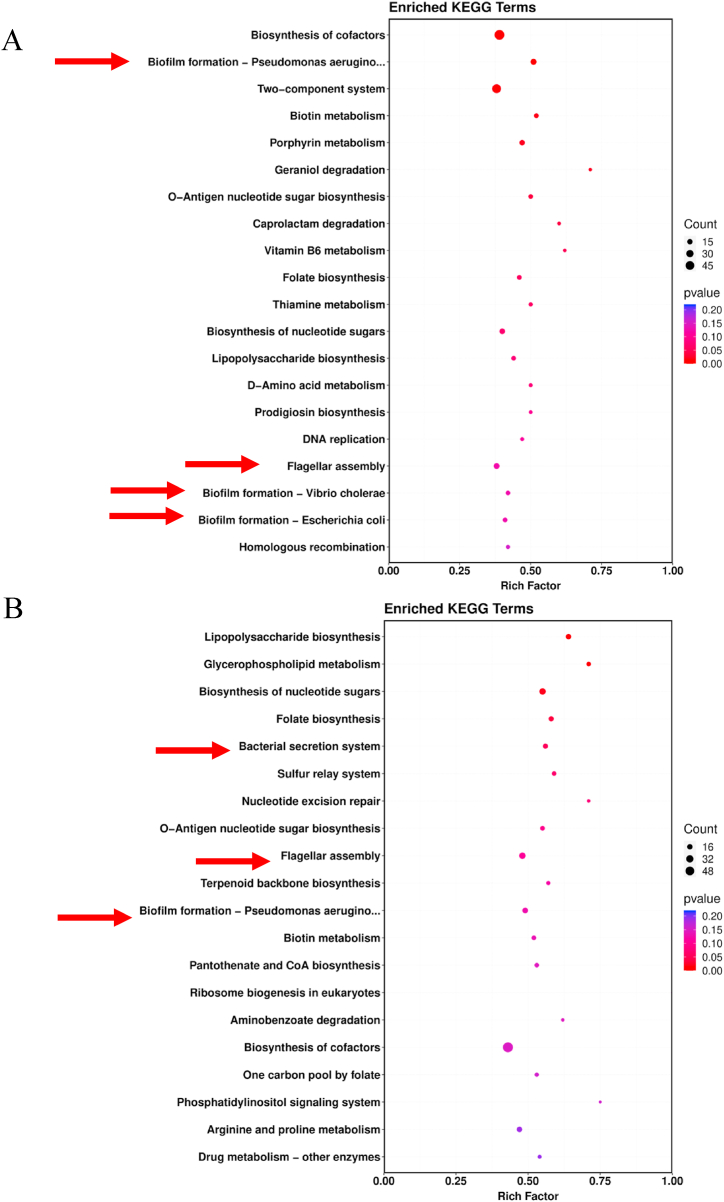
KEGG pathway-based enrichment analysis of the identified DSPs at 12 h (A) and 24 h (B). The enrichment of identified proteins against all KEGG database proteins was used to identify enriched pathways via two-tailed Fisher's exact tests. Pathways with corrected *p*-values <0.05 were considered significantly enriched.

We performed a KEGG analysis to determine the key enrichment pathways of DSPs by blasting against the online KEGG database (http://geneontology.org/). As shown in Fig. 3, the vast number of DSPs related to biofilm formation and flagella assembly pathway were enriched at 12 and 24 h, indicating that *popBD* knockout may affect these functions.

**Fig. 4:**
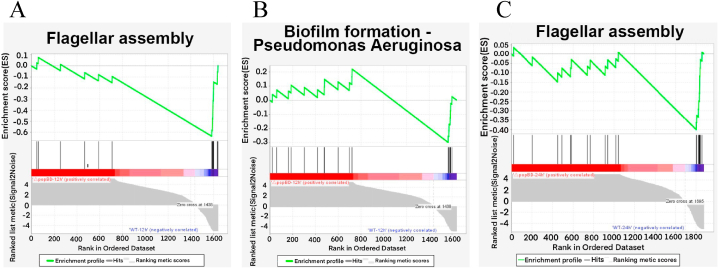
Gene Set Enrichment Analysis (GSEA) was used to identify gene sets that exhibited significant upregulated in the WT strains compared to *popBD* knockout group at 12 h (A and B) and 24 h (C). In the enrichment plot, the x-axis shows the rank order of genes from the most upregulated to the most downregulated between the WT and △*popBD* strains. The y-axis shows the distribution of the running enrichment score generated by walking down the list of ranked genes.

To further determine the coordinated secreted protein changes after popBD knockout in *P. plecoglossicida*, GSEA-based KEGG enrichment analysis was conducted. Compared with the △popBD group, the flagellar assembly and biofilm formation pathways were upregulated in the WT group (Fig. 4A and B, Supplemental Table 2). Similar to the 12 h GSEA pathway analysis, the upregulated pathway in the WT strain group was the flagellar assembly (Fig. 4C and Supplemental Table 2). All these data indicated that the popBD protein plays important roles in flagellar and biofilm formation.

### 3.4 popBD knockout affected the flagella-related gene expression and protein secretion

The effect of *popBD* knockout on protein secretion was also examined. The results show that proteins secretion was blocked in the mutant strains at two time points (12 and 24 h). Proteins present in the supernatant of the WT but absent in that of the mutant strain, whose abundance fold change was greater or equal to 5 and *p*-value less than 0.05, are listed in Table 2. A total of 39 proteins were significantly affected by *popBD* knockout. Of these proteins, flagella-related proteins (10/39) and the T3SS component (8/39) were the most abundant class, suggesting that there was a cross-talk between type III Secretion and flagella assembly.

**Table 2:** List of upregulated secreted proteins in the *P.plecoglossicida* WT versus △*popBD* mutant strains at both 12 and 24 h.

Protein ID	Gene name	Protein Name	12 h		24 h		Function
			FC	*P-* value	FC	*P-* value	
S2KST9	L321_12,104	Fis family transcriptional regulator	+/−*		+/−		
S2KL93	rhlB	ATP-dependent RNA helicase RhlB	+/−		+/−		
S2KR72	L321_00070	Secretion protein HlyD family protein	+/−		+/−		
A0A495PGE3	C7318_4817	DNA recombination protein RmuC	+/−		+/−		
S2KHW1	flgC	Flagella basal-body rod protein FlgC	+/−		47	2.1E-03	Flagella component
A0A2R7UHG9	cyoA	Ubiquinol oxidase subunit 2	+/−		19	1.1E-02	
S2KRP7	fliE	Flagella hook-basal body complex protein FliE	+/−		33	4.2E-03	Flagella component
S2K4A5	L321_14,276	Peptidoglycan-binding protein	+/−		10	3.3E-02	
S2KPG9	pscP	Translocation protein in type III secretion PscP	+/−		44	2.4E-03	T3SS component
S2K3G2	flgD	Basal-body rod modification protein FlgD	+/−		38	3.2E-03	Flagella component
S2K6D7	L321_09699	Lipoprotein	+/−		8	4.3E-02	
S2JVY3	exoU	Type three secretion system effector protein ExoU	392	2.0E-09	221	6.4E-05	T3SS component
S2JVT5	L321_18,312	Rhs element Vgr protein	1659	5.9E-13	619	3.9E-06	
S2KPH9	L321_18,482	psc I	1380	1.8E-12	65	1.1E-03	T3SS component
S2K109	L321_18,367	popD	589	2.3E-10	777	2.0E-06	T3SS component
S2KFH0	L321_18,372	popB	338	4.2E-09	1341	3.8E-07	T3SS component
S2JWH4	L321_17,907	2-methylaconitate *cis*-trans isomerase PrpF	298	7.8E-09	2840	3.2E-08	
S2KJR6	L321_11,795	Pyrroloquinoline quinone biosynthesis protein PqqE	1068	8.2E-12	20	1.0E-02	
S2KFH3	L321_18,397	hypothetical protein	467	8.0E-10	32	4.5E-03	Located within pcrD and pcrR
S2K134	pscF	pscF	439	1.1E-09	14	1.9E-02	T3SS component
S2KPF7	pcrV	Type III secretion protein PcrV	360	3.0E-09	31	4.6E-03	T3SS component
S2JY96	fliD	Flagella hook-associated protein 2 FliD	254	1.7E-08	11	2.7E-02	Flagella component
S2KHV4	flgG	Flagella basal-body rod protein FlgG	155	1.7E-07	21	9.3E-03	Flagella component
S2K105	hcp	Type VI secretion system tube protein Hcp	154	1.8E-07	16	1.6E-02	
S2JZ27	flgK	Flagella hook-associated protein 1	106	9.0E-07	72	8.6E-04	Flagella component
S2JZ33	flgF	Flagella basal-body rod protein FlgF	92	1.7E-06	62	1.2E-03	Flagella component
S2JY81	fliK	Flagella hook-length control protein	78	3.2E-06	24	7.5E-03	Flagella component
S2KP56	L321_17,912	Aconitate hydratase	57	1.2E-05	19	1.1E-02	
S2KHU7	flgL	Flagella hook-associated protein FlgL	42	3.5E-05	39	3.1E-03	Flagella component
S2K564	tsaD	tRNA N6-adenosine threonylcarbamoyltransferase	39	2.4E-03	19	1.0E-02	
S2KQD5	yidC	Membrane protein insertase YidC	28	1.4E-04	90	5.3E-04	
S2JYB3	flgE	Flagella hook protein FlgE	27	1.6E-04	9	3.6E-02	Flagella component
S2JTF5	L321_21,987	Translation initiation factor SUI1	26	5.6E-03	26	6.3E-03	
S2K088	L321_19,752	CpsD/CapB family tyrosine-protein kinase	15	1.6E-02	75	7.9E-04	
S2K4G6	L321_05794	Sel 1 repeat family protein	10	3.7E-03	15	1.8E-02	
S2KRC2	secF	Protein-export membrane protein SecF	8	5.4E-03	44	2.5E-03	
A0A495P7C9	C7318_1445	VCBS repeat-containing protein	8	6.8E-03	87	5.8E-04	
S2K1N0	hsp 20	Heat shock protein Hsp20	8	4.4E-02	44	1.9E-03	
S2KQS0	L321_00687	Wzz domain-containing protein	5	1.8E-02	58	1.4E-03	

Note: * ± indicating the protein identified in the WT group while absent in the mutant group.

**Table 3:** Primers used in this study.

Gene name	Primer sequence (5′-3′)
18Tc-Δ*popBD*-up F	tgattacgaattcgagctcgGCACACTGACACACGAGGTG
18Tc-Δ*popBD*-up R	TGAACGATGAGGATTCAGACGCCCAGCC
18Tc-Δ*popBD*-down F	GTCTGAATCCTCATCGTTCAATGCTCCTCAGT
18Tc-Δ*popBD*-down R	taaaacgacggccagtgccaTGGTTATACCAGCGCTGAACAG
Δ*popBD*-external F1	AGTGCTGCAACAAATCGCG
Δ*popBD*-external R1	CACTGAAATCCTTCAGGGGCAG
Δ*popBD*-internal F2	ATGATACCTTGCCCAGCTGC
Δ*popBD*-internal R2	CGGTGATGATGGCAGTGGAG
flagella basal body *P*-ring formation protein FlgA F	CACCCTGGACGTTGTTCGTA
flagella basal body *P*-ring formation protein FlgA R	CACGTCGTTTTCGCCAATCA
flagella basal body rod protein FlgB F	CATCCACGAAAAAGCCCTGG
flagella basal body rod protein FlgB R	ATGTCACGCGCCTTGTAGTT
flagella basal body rod protein FlgC F	GTGTCTTCCATATCGCCGGT
flagella basal body rod protein FlgC R	TGGAAGGTAGTGGCGAACAC
flagella hook assembly protein FlgD F	TCGAAGGCATCACCAACCTC
flagella hook assembly protein FlgD R	GGTTTGTACGTCGGAGAGGG
flagella hook protein FlgE F	GACCAGGGCATCAACCTCAA
flagella hook protein FlgE R	TGTAGGTGGTCTCGTCGGAT
flagella hook protein FlgF F	CGGTCTGATCCACACCGAAA
flagella hook protein FlgF R	GCCGCGTTCATCATCTTCAC
flagella basal-body rod protein FlgG F	AGCAGACCCTGGAAAACTCC
flagella basal-body rod protein FlgG R	GAACGAAAGCATCTGGTCGG
flagella basal body L-ring protein FlgH F	AACGGTTCGATCTACCAGGC
flagella basal body L-ring protein FlgH R	AGGTCTTCTCGTTGAGCGTG
flagella basal body *P*-ring protein FlgI F	GTTTCAACCAGGGCAACACC
flagella basal body *P*-ring protein FlgI R	ACCTTGACGTTCTGGCCAAT
flagella assembly peptidoglycan hydrolase FlgJ F	GGAGTTCGAGTCGCTGTTCA
flagella assembly peptidoglycan hydrolase FlgJ R	TGACCGTCGAGCTGTTCATC
flagella hook-associated protein FlgK F	CTGGACATCTACGACTCGGC
flagella hook-associated protein FlgK R	GTGAAGCTGTCGCCGTTTTT
flagella hook-associated protein FlgL F	CTGCAGGTAGGCGACATGAT
flagella hook-associated protein FlgL R	GAAATCTCGTCGGCCTTGGA
flagella biosynthesis anti-sigma factor FlgM F	GCAGTTGCAGAAGATCAACGA
flagella biosynthesis anti-sigma factor FlgM R	GAGCTTCGAAATCGAGCAGTT
flagella protein FlgN F	TGCCAGAAGGTCAACGAGAC
flagella protein FlgN R	CGGCTGTCGTAGAGAGATGG
flagella filament capping protein FliD F	AACACCATCGAGAATGCCGT
flagella filament capping protein FliD R	ACAACTCCTGCACTTCCGAG
flagella hook-basal body complex protein FliE F	TCGACCTGACCGATGTGATG
flagella hook-basal body complex protein FliE R	TCTGCATGATGTCCTGGTACG
flagella M-ring protein FliF F	TCATGGAAGCCACCCGTTAC
flagella M-ring protein FliF R	ACACCGAGCTTTTCGGGATT
flagella assembly protein FliH F	AGACCTTGCACAGCCGTATC
flagella assembly protein FliH R	AAAGACGTCCAGGTCGATGG
flagella protein export ATPase FliI F	GGCAAGAACGTGTTGCTGTT
flagella protein export ATPase FliI R	TAATGGCCTTCTTCGGCCAG
flagella biosynthesis chaperone FliJ F	GCTGGCTGCTCAACTACCA
flagella biosynthesis chaperone FliJ R	CTCGGGCGTTCTTGAGGTT
flagella hook-length control protein FliK F	AAGTAGCCGATGCATCCCAG
flagella hook-length control protein FliK R	TATCGCTACTCGCGCTCTTG
flagella basal body-associated protein FliL F	TGTCATGCACAAGAGCGAGT
flagella basal body-associated protein FliL R	ATCACCAGGTTGTTGCGGAT
flagella motor switch protein FliM F	AGTGCTTCGTCGACCTCAAG
flagella motor switch protein FliM R	CATCGAGTACGGCATGGTCA
flagella motor switch protein FliN F	GCTCTGGAAGAAACCGGTGA
flagella motor switch protein FliN R	GGATCACATCCAGGTTCGGG
flagella biosynthetic protein FliO F	TGGTCGGGCTGATCTTCTTTC
flagella biosynthetic protein FliO R	CTGCCGACGATCTCGATCAC
flagella type III secretion system pore protein FliP F	CACCTCGGAACTGAAGACGG
flagella type III secretion system pore protein FliP R	GCATGATCTTGAACGGCAGC
flagella biosynthesis protein FliQ F	GGTGATGCTGGTGACACTGA
flagella biosynthesis protein FliQ R	GTACAGGCCGGTGATGTACT
flagella type III secretion system protein FliR F	GCTGATGACCATGCCGATCT
flagella type III secretion system protein FliR R	GCAGGGAAAACCCGAACAAC
flagella export chaperone FliS F	CCATGCTGAAAGTTTGGCCG
flagella export chaperone FliS R	GGTCGCTATTCACGTTGGCT
flagella protein FliT F	CTGCAGGTCTACAGGCAACT
flagella protein FliT R	ACGGATTTGCGTCATCTCGT
rpoD F	AGAGTCCGAAAGCAGTGACG
rpoD R	CGCTCGACCAGTACATCGAA

**Fig. 5:**
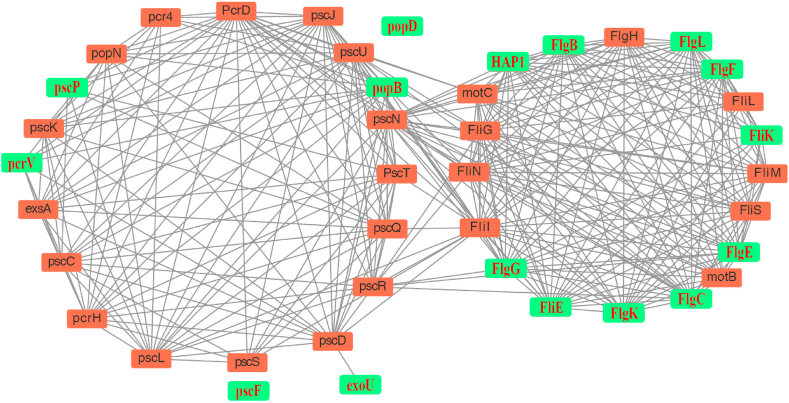
Protein-protein interaction network of T3SS and flagella-related proteins. Label with red font over green background represents differential secreted proteins between △*popBD* and WT strains.

We further constructed the PPI network of all the annotated T3SS-related proteins and flagella-related proteins of *P. plecoglossicida*. Many T3SS- or flagella-related DSPs identified in this study, labeled with red font over a green background, are interlinked (Fig. 5).

**Fig. 6:**
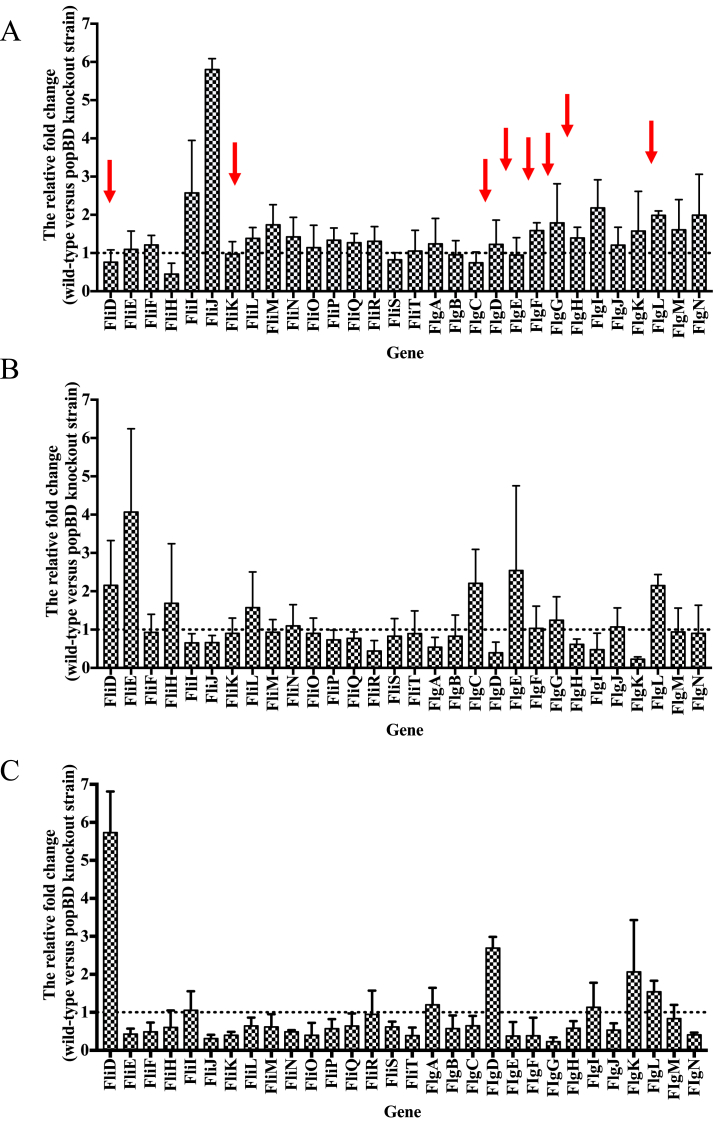
Effect of *popB* and *popD* knockout mutations on the expression of flagella-related genes at 6 h (A), 12 h (B) and 24 h (C). Expression values for each gene from the different groups were normalized to the rpoD transcript levels, which remain constant regardless of changes in experimental conditions. The fold change was determined as the relative expression of genes from the WT strain relative to the mutant strain using the 2^−ΔΔCt^ method. The data are presented as means of three replicates. Error bars represent standard deviations.

Next, we compared the levels of expression of these flagella-related genes between WT and *popBD* knockout mutant strains. Compared to the WT strain, the flagella-related genes expression was repressed in the *popBD* knockout strain to varying degrees at the initial stage (6 h), except *fliD, fliH, fliS,* and *flgC* which were slightly upregulated (Fig. 6A). At 12 h, expression of seven genes, including *fliD, fliE, fliH, fli, flgC, flgE* and *flgL,* was down-regulated compared with WT strain. At the same time, some genes (*fliR, flgA, flgD, flgH, flgK and flgL*) were upregulated (Fig. 6B). At 24 h, most of the flagella-related genes expressions was repressed in the WT strain. However, the *fliD, flgD, flgI,* and *flgK of* WT strain was upregulated (Fig. 6C).

Interestingly, among the 10 flagella-related genes listed in Table 2, 5, 5 and 4 of 10 genes were up-regulated in the WT strains at 6, 12, and 24 h, respectively (Red arrow). Altogether, these results suggested that *popBD* knockout might impact the flagella-related protein secretion by regulating the expression of genes shown in Table 2. However, the mechanisms leading to upregulation of these genes are unknown and, therefore, warrant further investigations.

### 3.5 The influence of popBD knockout on P. plecoglossicida motility, adhesion, and biofilm formation

**Fig. 7:**
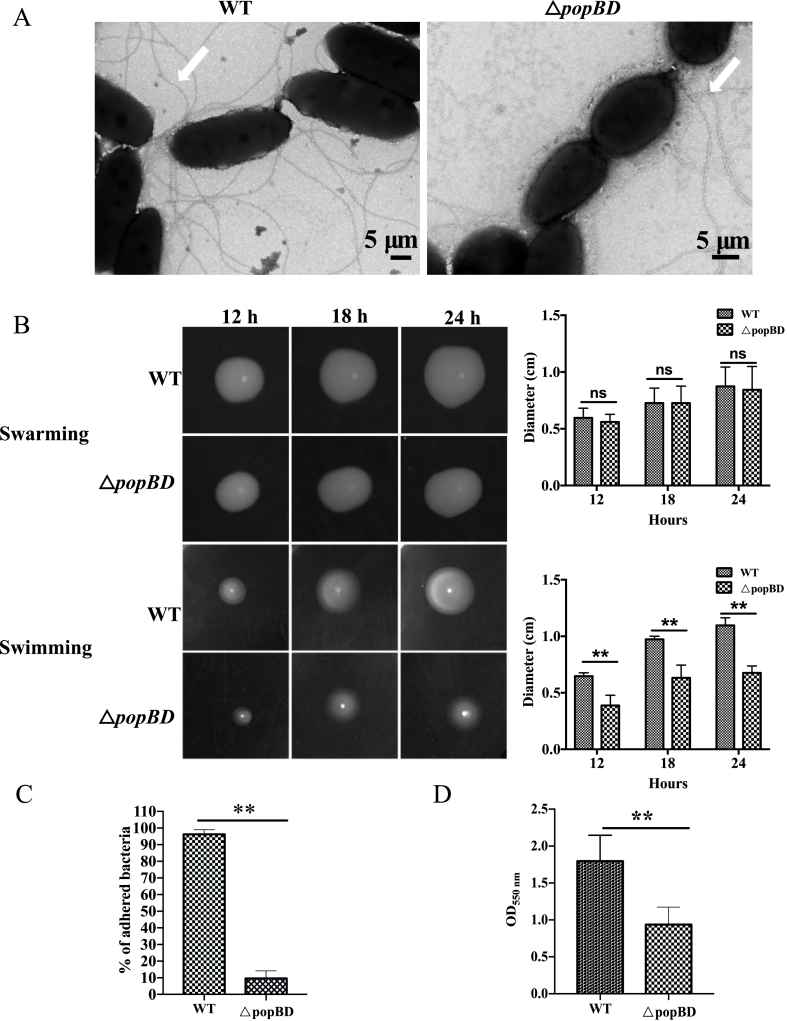
Phenotypes of the *P. plecoglossicida WT* and △*popBD* mutant strains. (A). Electron micrograph of the WT (note presence of multiple flagella around bacteria cells) and mutant strain (note presence of extremely fewer or absence of flagella around bacteria cells) ( × 8000); (B). *P. plecoglossicida* WT and mutant strains were plated on swarming or swimming motility agar and incubated at 28 °C for 12, 18, and 24 h. Images shown are representatives from independent experiments (left). Diameters across the zones of bacterial swimming or swarming were measured and the means and standard errors of the means for all three experiments (right). The data are presented as means of three replicates. Error bars represent standard deviations. (C) Adherence of *P. plecoglossicida* WT and △*popBD* mutant strains onto EPC cells. (D) Biofilm formation of *P. plecoglossicida* WT and △*popBD* mutant strains measured by CV assay. All data were processed using SPSS software (version 20.0) and Graphpad Prism program. Student's t-tests were used to evaluate the statistical significance. Statistical significance was set to a *p*-value of 0.05.

The Δ*popBD* mutant, alongside the WT strain, was tested for the phenotypic properties related to flagella. Firstly, the impact of *popBD* knockout on the flagella morphology was studied using transmission electron microscopy (TEM) (Fig. 7A). The results show the number and length of the flagella filaments of the mutant strain were significantly lower than the WT strain.

Bacterial adhesion, motility, and biofilm formation are phenotypic properties known to influence pathogenesis. According to our proteomics results, *popBD* knockout impacted the flagella assembly- and biofilm formation-related pathway. The effect of the *popBD* mutations on swarming motility, cell adhesion, and biofilm formation in *P. plecoglossicida* have also been investigated. Different strains of *P. plecoglossicida,* including the △*popBD* mutant and the WT strain, were plated on the swarming agar or swimming agar. Swarming motility is characterized by movement across a semisolid surface, which requires flagella motility and the production of biosurfactants. While swimming motility is defined as the movement in liquid or low-viscosity conditions (up to 0.3 % agar concentration). Swimming motility, which different from swarming motility, requires the functional flagellum, but neither quorum sensing (QS) systems nor biosurfactants. The results showed that there was no significant difference in swarming motility between *popBD* knockout and WT strain at 12, 18, and 24 h (Fig. 7B, up). However, the mutant strain exhibited reduced swimming when compared to the WT (Fig. 7B, down). We further compared the adherence ability of *P. plecoglossicida* WT and mutant strain to Epithelioma Papulosum Cyprini (EPC) cells. Based on the number of bacteria that adhered to EPC cells, the percentage of adhered *P. plecoglossicida* WT to the amounts of bacteria initially incubated with EPC cells was 96.2 %. Adherence of △*popBD* to EPC cells was markedly reduced compared with the WT strain. The adherence rate of the mutant strain was 9.6 % (Fig. 7C). To analyze the effect of *popBD* knockdown on biofilm formation phenotypes, the quantitative crystal violet (CV) was conducted. The results showed that the Δ*popBD* strains possessed weaker biofilm-forming ability at all stages of growth (Fig. 7D).

These observations above revealed that popBD protein might negatively affect the function of the flagellum.

## 4 Discussion

In this study, we have shown that deletion of the T3SS translocon significantly altered the profile of T3SS and flagella proteins secreted by *P. plecoglossicida*. We have also shown that the *P. plecoglossicida* Δ*popBD* mutant strain, compared to the WT strain, produced different phenotypic properties related to flagella such as the flagella morphology/formation, adherence, mobility, and biofilm formation. Altogether, our findings point to a cross-talk between the T3SS machinery and flagella assembly in *P. plecoglossicida.* This observation was supported by data from the PPI network analysis, which showed an interlinkage pointing to a cross-talk between T3SS and flagella proteins. Our results, which are in agreement with previous studies, showed a direct overlap between changes in the T3SS machinery and flagella assembly in other bacteria species. The T3SS regulator ExsA functions directly or indirectly in repressing the expression of the lateral flagella system in *Aeromonas hydrophila* [46] and *Vibrio parahaemolyticus* [47]. Compared to wild-type *P. aeruginosa*, mutants deficient in flagella and motility produced increased amounts of T3SS needles and secreted abundant effectors [48]. Conversely, the overproduction of ExsA down-regulates the flagella regulon's expression and decreases the bacterium's mobility [49]. These observations suggest a reciprocal cross-control in the two systems at the transcriptional level. A study recently revealed that enteropathogenic *Escherichia coli* (EPEC) T3SS basal components knockout (Δ*espA* mutant) results in the inability to form flagella. The translocon or effector-deficient mutants were significantly diminished in flagella production [50]. The ExoU is a T3SS effector whose secretion can be indicative of a functional T3SS. In this study, we found that the ExoU and other T3SS component protein secretion significantly decreased in the Δ*popBD* mutant strain, suggesting that *popBD* knockout disrupts the function of T3SS. As the key components of flagella assembly, such as the basal flagellum body, rod, integral component of the flagella membrane, and flagellum hook, were also strongly diminished in the Δ*popBD* mutant strain, we speculated that flagella components might also be substrates of the T3SS. This is in line with Dilek et al. who found that FliD, FlgK, FlgL, FlgE, and FlgB are secreted more efficiently by the T3SS than by the flagellum in *P. aeruginosa* [51]*,* suggesting that this phenomenon could be conserved among the Pseudomonads. As we know, rod, hook, hook-associated, cap, and flagellin proteins are secreted and assembled in a highly ordered process during flagellum biosynthesis, [52]. Thus, we speculated that the repression of flagella seen in the T3SS mutants is at the post-transcriptional level, and the fewer and shorter flagella filaments observed in the Δ*popBD* mutant might be caused by abnormalities in flagella component translocation.

Several studies show that translocon proteins play an important role in preventing the leakage of cytoplasmic proteins into the extracellular compartment [26,28]. This implies that deletion of *popB* and *popD* could lead to secretion of cytoplasmic proteins into extracellular compartment. In the present study this was evaluated by quantifying the cytoplasmic proteins released in the growth media used for the culture of the *P. plecogossicida* WT and Δ*popBD* mutant strains. Thus, the presence of more cytoplasmic proteins in growth media used for culture of the Δ*popBD* strain than in the WT strain is indicative that deletion of *popB* and *popD* proteins enhanced extracellular leakage of cytoplasmic proteins in growth media. This finding corroborates with Hauser et al. [32] who found deposition of extracellular protein aggregates in mutant strains of *P. aeruginosa* with defective T3SS translocon proteins and Parsot et al. [31] who also showed that defective T3SS translocon proteins in Shigella flexneri led to secretion of cytoplasmic proteins extracellular. Thus, our findings concur with previous studies showing that defective translocon protein lead to secretion of cytoplasmic proteins outside host cells into extracellular compartments.

Bacterial attachment to surfaces is mediated, in most cases, by swimming and swarmming. Swarming is a collective movement of the bacterial population on semisolid agar [53] whereas the locomotion characteristics of swimming are mediated by the number of flagella, their arrangement on the cell body, and their sense of rotation [54]. Our observation shows that the low swimming ability observed in the Δ*popBD* strain corresponded with the poor flagella formation observed by TEM examination, suggesting that the deformity in flagella formation linked to *popB-popD* deletion could have altered the swimming ability of the Δ*popBD* mutant strain. Our findings show that the Δ*popBD* mutant had the same swarming properties as the WT strain indicating that deletion of the popB-popD proteins did not alter the swarming ability of *P. pecoglossicida* on the semi-solid agar. The specific mechanisms responsible for the differences should be further investigated in forthcoming studies. We also observed that the low adherence ability on EPC cells corresponded with the weak biofilm formation ability in the Δ*popBD* mutant, whereas the high adherence ability corresponded with high biofilm formation in the WT strain. Thus, it can be concluded that although both the Δ*popBD* mutant and WT strains had similar swarming abilities, the WT strain had a higher adherence and biofilm formation capacity than the Δ*popBD* mutant. These observations are in line with Murray et al. [55] who found a negative association between swarming and biofilm formation in 237 *P. pseudomonas* isolates from hospitalized patients. Given that flagella are important adherence proteins used for attachment to host cells during the early stages of bacterial infection [56], it is likely that alteration of flagella formation by PopB-PopD protein deletion can lead to the reduction of virulence in *P. plecogossicida* infections in fish. Equally, the weak biofilm formation ability of the Δ*popBD* mutant entails that it is likely to be less virulence than the WT strain, given that biofilm formation is an important virulence factor in Gram-negative bacteria [57]. Altogether, the low adherence and weak biofilm formation seen in the Δ*popBD* mutant points to the loss of virulence, which could render this strain to serve as an attenuated vaccine candidate against *P. plecogossicida* infections in fish. Thus, future studies will seek to determine whether it could be protective against the WT strain.

## 5 Conclusions

In the present study, fewer and shorter flagella filaments were observed by TEM in the Δ*popBD* mutant, compared with the WT strain that had normal long flagella filaments, suggesting that deletion of the *popB-popD* genes altered the flagella assembly in *P. plecogossicida*. A comparison of expression levels of flagella-related genes in the Δ*popBD* mutant and the WT strains showed significantly lower expression levels of flagella-related genes in the Δ*popBD* mutant than in the WT strain. Thus, it is likely that the low expression levels of flagella-related genes such as the flagella basal body, rod, and hook protein genes in the Δ*popBD* mutant detected in the GO and KEGG pathway analysis were linked to the low presence of flagella filaments. The flagella filaments in the Δ*popBD* mutant were mostly short and impaired whereas the normal long filamentous flagella were abundantly seen in the WT strain by TEM, which was likely due to higher expression levels of flagella-related genes in the WT strain. Overall, our findings point to a cross-talk between the T3SS machinery and flagella assembly in *P. plecoglossicida*.

## Data availability

The mass spectrometry proteomics data have been deposited to the ProteomeXchange Consortium via the PRIDE partner repository with the dataset identifier PXD042864.

## CRediT authorship contribution statement

**Pan Qin:** Writing – original draft, Methodology, Investigation, Data curation, Conceptualization. **Yingjia Luan:** Methodology, Investigation. **Jinmei Yang:** Methodology, Investigation. **Xingfu Chen:** Methodology, Investigation. **Tong Wu:** Methodology, Investigation. **Yousheng Li:** Methodology, Investigation. **Hetron Mweemba Munang'andu:** Writing – review & editing. **Guangming Shao:** Data curation, Conceptualization. **Xinhua Chen:** Writing – review & editing, Conceptualization.

## Declaration of competing interest

The authors declare that they have no known competing financial interests or personal relationships that could have appeared to influence the work reported in this paper.
