# Supplementary figures and images for: Comparative secretome analysis reveals cross-talk between type III secretion system and flagella assembly in Pseudomonas plecoglossicida
Source: Heliyon. 2023 Dec 2;9(12):e22669. doi: 10.1016/j.heliyon.2023.e22669 (PMC10746435; doi:10.1016/j.heliyon.2023.e22669)

## Slide 1
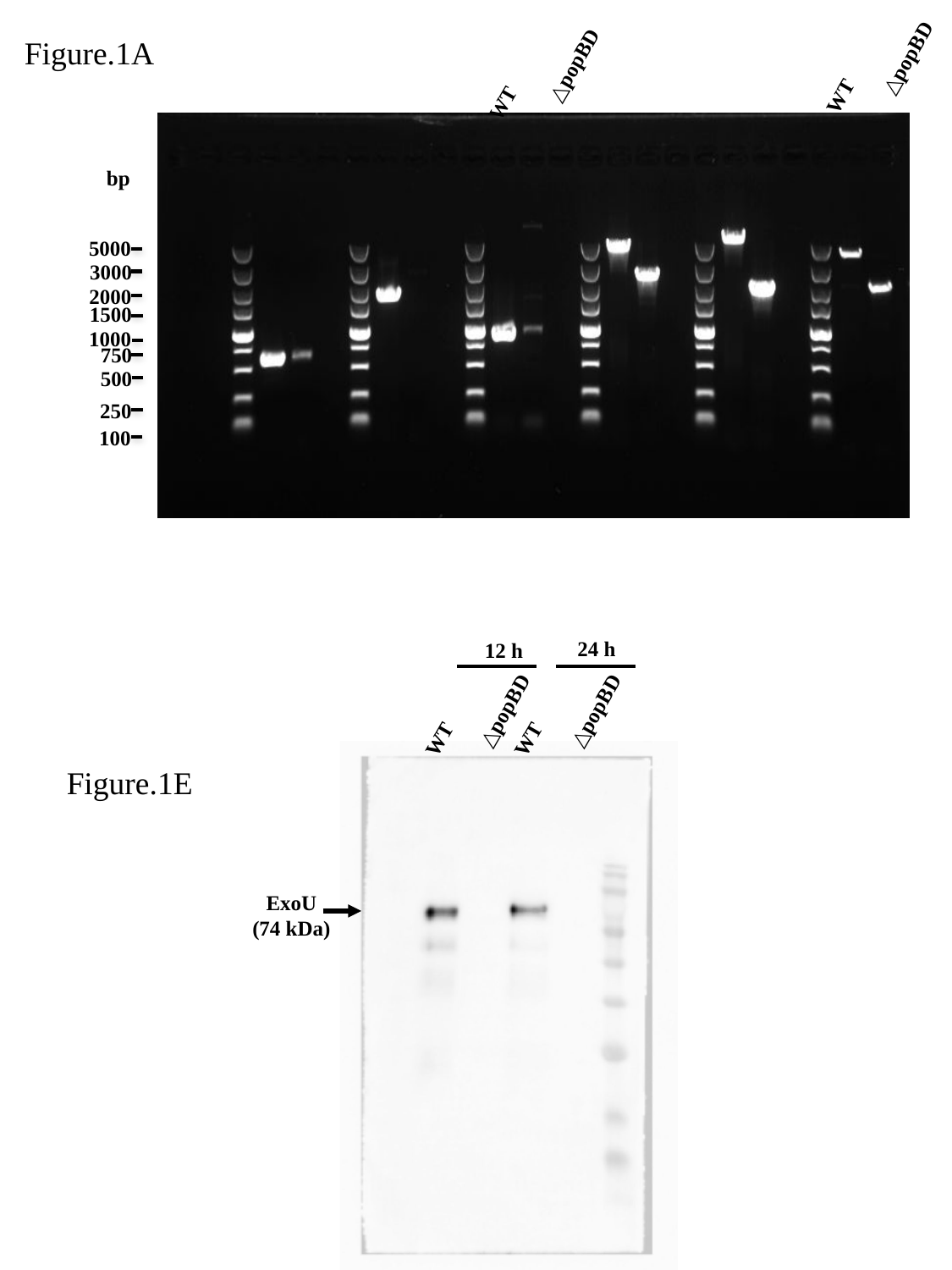

Figure.1A
△popBD
△popBD
WT
WT
bp
5000
3000
1500
1000
750
500
250
100
2000
24 h
12 h
△popBD
△popBD
WT
WT
Figure.1E
ExoU
(74 kDa)

Supplement: Multimedia component 1 [file mmc1.pptx]
